# The Impacts of Sports Schools on Holistic Athlete Development: A Mixed Methods Systematic Review

**DOI:** 10.1007/s40279-022-01664-5

**Published:** 2022-03-09

**Authors:** Ffion Thompson, Fieke Rongen, Ian Cowburn, Kevin Till

**Affiliations:** 1 Carnegie School of Sport, Leeds Beckett University, Leeds, UK; 2 Queen Ethelburga’s College, Thorpe Underwood, York, UK; 3 Leeds Rhinos Rugby League Club, Leeds, UK; 4 Carnegie School of Sport, Leeds Beckett University, Room G07, Cavendish Hall, Headingley Campus, Leeds, LS6 3QS UK

## Abstract

**Background:**

To understand the multiple and wide-ranging impacts of intensified youth sport, the need for a holistic approach to athlete development has recently been advocated. Sports schools are an increasingly popular operationalisation of intensified youth sport, aiming to offer an optimal environment for holistic development by combining sport and education. Yet, no study has systematically explored the impacts associated with sports schools.

**Objectives:**

The aims of this mixed method systematic review were to (1) determine the characteristics and features of sports schools; (2) identify the methods used to evaluate sports school impacts, and (3) evaluate the positive and negative holistic athlete development impacts associated with sports school programme involvement.

**Methods:**

Adhering to PRISMA guidelines, eight electronic databases were searched until the final return in February 2021. Forty-six articles satisfied the inclusion criteria, were analysed thematically, and synthesised using a narrative approach. The methodological quality of included studies was assessed using the Mixed Methods Appraisal Tool.

**Results:**

Findings indicated (1) sports school student-athletes receive considerable support in terms of academic and athletic services, more intensified training and competition schedules with high-level training partners, but regularly miss school; (2) multiple methods have been used to evaluate student-athlete impacts, making comparison across studies and developing consensus on the impacts of sports schools difficult; and (3) there are a multitude of immediate, short- and long-term positive and negative impacts associated with the academic/vocational, athletic/physical, psychosocial and psychological development of sports school student-athletes.

**Conclusions:**

This study is the first to systematically review the research literature to understand the impacts associated with sports schools in terms of holistic athlete development. Practitioners should be aware that they can promote (positive) and negate (negative) health impacts through the design of an appropriate learning environment that simultaneously balances multiple training, academic, psychosocial and psychological factors that can be challenging for youth athletes. We recommend that practitioners aim to design and implement monitoring and evaluation tools that assess the holistic development of student-athletes within their sports schools to ensure they are promoting all-round and healthy youth athlete development.

## Key Points

**Table:** 

Sports school student-athletes receive more support in academic and athletic services than non-sports school athletes.
There are a multitude of immediate, short- and long-term positive and negative impacts associated with being a sports school student-athlete that stakeholders should be aware of when designing, implementing, and evaluating sports school programmes.
Practitioners should aim to design and implement monitoring and evaluation tools that assess the holistic development of student-athletes within their sports schools to ensure they are promoting healthy youth athlete development.
The large range of data collection methods used to evaluate the impacts of sports school programmes makes comparison across studies difficult but offers multiple avenues for future research.

## Introduction

The present-day outlook of Olympic and professional sport is now arguably more competitive than ever. One consequence is the increased intensity and professionalisation of youth sport programmes supporting athletes towards the Olympic and professional level [1, 2]. This increased professionalisation of youth sport programmes introduces a number of characteristics, such as early specialisation [3], increased volume and intensity of training [4], prioritisation of sports over other aspects of life [5], and distinct cultures of eliteness [6], raising potential issues with the healthiness of intensified youth sports programmes. Indeed, recent position and consensus statements [1, 7] have warned about the risk of several negative impacts associated with intensified youth sport programme involvement. Rongen et al. [8] emphasised that there are also potential positives but that ensuring healthiness may require a balancing act. For the purpose of this paper, impact is not confined to outputs (athletic performance), but also incorporates the holistic development of youth athletes. As such, potential impacts include academic/vocational (e.g., academic high achievers vs. educational sacrifice), athletic/physical (e.g., enhanced physiological capacity vs. injury), psychosocial (e.g., time away from family vs. enhanced social skills, such as communication), and psychological (e.g., increased confidence vs. burnout) areas. Given their popularity, the likelihood that most youth athletes do not ultimately succeed in their sport, and the multiple and wide-ranging positive and negative impacts associated with intensified youth sport programmes, understanding the holistic development impacts for youth athletes in these programmes is crucial [8, 9] to ensure the promotion of healthy development.

In light of the multiple and wide-ranging potential impacts of intensified youth sport programmes, the need for a holistic approach to an athlete's development has recently been advocated [10–13]. In response to these calls, researchers have increasingly followed Wylleman’s [14] Holistic Athletic Career model where for healthy, all-round development, youth sport programmes should embrace the multidimensional nature of youth athlete development. As conceptualised by the Holistic Athletic Career model [14], throughout their sporting careers there are constant interactions between all levels of an athlete's development (e.g., academic/vocational, athletic/physical, psychosocial and psychological). This means that transitions occurring in one domain (e.g., athletic development) are concurrent and interact with transitions occurring in another domain of an athlete’s life (e.g., academic studies). Therefore, although practitioners may instinctively focus on assessing and monitoring measures of physical performance, for the holistic development of youth athletes, it is imperative that considerations are also given to the academic/vocational, psychosocial and psychological domains [14].

By advocating a holistic approach, youth sport programmes are not only nurturing successful athletes but also developing competencies and skills that allow them to cope with challenges they face both in sport and other life domains. More specifically, to ensure this healthy all-round development and minimise the potential negative impacts of intensified youth sport programmes highlighted above, a dual-career approach to athlete development has been encouraged. This proposes that youth athletes must successfully develop their athletic career alongside pursuing education and/or vocation, and other domains (e.g., social life [10, 13, 14]). Indeed, the combination of sport and education or vocational endeavours has been shown to have benefits such as improving coping with adversity, protecting against poor mental health or burnout, and maintaining perspective for athletes [15–18]. However, the way dual-career development environments (i.e., environments that support dual-career athletes [19]) are shaped and the support provided is highly variable [17, 20, 21].

One example of a dual-career development environment that aims to cater for the holistic development of youth athletes is a sports school. A sports school is defined as a school, whether state-funded or private, that concentrates resources on developing sporting talent either within the curriculum and/or through extra-curricular activities [22–24]. Sports schools aim to safeguard the dual-career of school-aged athletes. In most countries, sports schools were founded in the early 1990s; however, sports boarding schools existed in the Soviet Union since 1962 [25]. While in some countries sports schools are part of a national sport system and in other countries they are not, all schools cater for elite student-athletes in systematic ways [26]. Attendance at these sports schools is voluntary and specific to the individual, school and sport context. In some contexts, the schools are state funded, in others students can be fee paying or receive a scholarship. Sports schools provide a structural coupling of competitive sports and education, accomplished by organising more time for training alongside sufficient time devoted to education [22]. For example, timetables can be adjusted by school officials to enable early training, allow exemptions from lessons for training and competition, and provide compensatory lessons [22, 24, 27, 28]. With the effective combination of competitive sports, education, and accommodation, sports schools could guarantee conditions that favour future top sporting performances while safeguarding opportunities for primary and secondary education [22] alongside allowing for more ‘free time’ through optimised time-schedules. Furthermore, many sports schools have specialist staff (e.g., physiotherapists, strength and conditioning coaches) [24] that may further support the holistic and healthy development of youth athletes.

Despite sports schools offering an optimum environment where positives could be maximised and negatives minimised, to date no study has attempted to systematically review the research literature to understand the impacts associated with sports schools in terms of holistic athlete development. Understanding the impacts associated with sports school involvement is important to inform the design, implementation, monitoring and evaluation of sports school programmes. Furthermore, there are many ways in which sports school systems can be implemented. Consequently, we need to understand the characteristics and features of such sports schools and how these relate to holistic athlete development impacts. Finally, there are multiple data collection methods/instruments to assess impacts and it would be beneficial to gain an understanding of the commonly used methods to guide future research. Therefore, the aims of this systematic review were to (1) determine the characteristics and features of sports school programmes; (2) identify the methods used to evaluate sports school impacts; and (3) evaluate the common positive and negative holistic athlete development impacts associated with sports school programme involvement.

## Methods

### Design and Search Strategy

A systematic review was conducted according to the Preferred Reporting Items for Systematic Reviews and Meta-Analyses Protocol (PRISMA-P) guidelines [29]. Adhering to PRISMA guidelines, a systematic search of eight electronic databases (The Cochrane Library, ERIC, PsycINFO, PsycArticles, PsycTESTS, SAGE Journals Online, Scopus and Academic Search Complete) was conducted to identify original research articles from the earliest available records up to and including January 2021 (when the formal search was finalised). Boolean search phrases were used to include search terms relevant to student-athletes (population; “Student-athlete”, “School student”, “Adolescent”, “Youth”, “Young”, “Junior”, “Elite”, and “Talented”) and the educational systems/types of sports school intervention; (“Sport School”, “Elite School of Sport”, “Topsport Talent School”, and “Dual Career”). Relevant keywords for each search term were determined through pilot searching (screening titles/abstracts/keywords/full texts of previously known articles). Keywords were combined within terms using the 'OR' operator, and the final search phrase was constructed by combining the two search terms using the ‘AND’ operator. Additional records were taken from the bibliographies of eligible studies and previous reviews. Attempts were made to contact two authors of the selected articles to request any missing relevant information. One author replied to confirm that participants were from a sports school sample.

### Study Selection

Duplicate records were identified and removed before the remaining records were screened against predefined inclusion–exclusion criteria (Table 1). Studies were screened independently by two researchers (FT, FR). The screening of the journal articles was completed over two phases. Studies were initially excluded based on the content of the titles and abstracts, followed by a full-text review. In the event of disagreement over the reviewer's decision, reviewers met to come to an agreed decision on the paper.

**Table 1:** Inclusion/exclusion criteria (title/abstract screening and full screening)

Criteria	Inclusion	Exclusion
1	Original peer reviewed research article	Reviews, surveys, opinion pieces, books, periodicals and editorial
2	Published in the English Language	Non-English publications
3	Published before 1/02/2021 (when the formal search was finalized)	
4	Population—Explicitly related to current or former primary or secondary sport school student-athletes	Primary or secondary non-sport school athletes
5	Either contains entirely sport school athletes or a separable discrete sports school athlete sample (e.g., comparing sport school athletes to non-sport school athletes)	University cohort and athletes with a physical or mental disability
6	Include data relevant and compatible with the study aims	Data not relevant or compatible with the study aims

### Search Returns

**Fig. 1:**
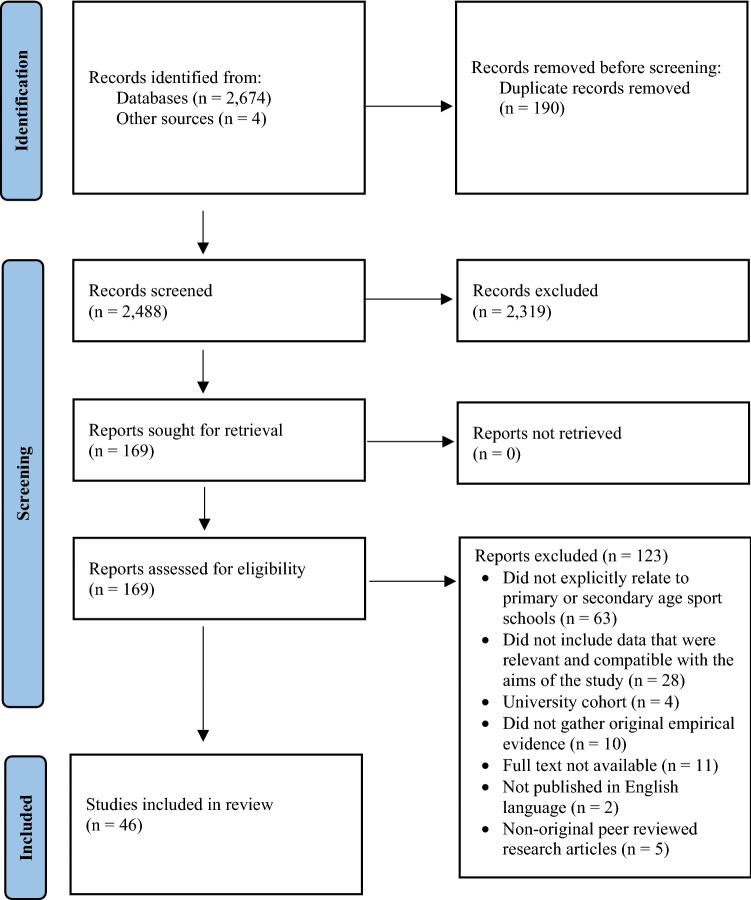
Flow of selection process of eligible studies

The final search phase was completed on 1 February 2021, and returned 2,488 studies following the removal of duplicates. After abstract screening against the inclusion/exclusion criteria, 2,319 papers were excluded, leaving a total of 169 studies. After each paper's full text was assessed against the inclusion/exclusion criteria, 123 papers were excluded due to not explicitly relating to primary or secondary age sports schools (*n* = 63), data irrelevant or not aligned to study aims (*n* = 28), full text was not available (*n* = 11), lack of empirical data (*n* = 10), published in non-English (*n* = 2), university cohort (*n* = 4) and non-original peer-reviewed research articles (*n* = 5). Therefore, a total of 46 papers met the inclusion criteria. The process of study identification, screening, and selection is presented in Fig. 1.

### Data Extraction

One author (FT) extracted the data using a specifically designed standardised Microsoft Excel spreadsheet. General information (i.e., author, year, country, and setting), study aim(s), study design, study population, data collection method and instrument, and the key findings presented in accordance with this systematic review’s aims were extracted.

### Quality Assessment

All included studies were critically appraised by two researchers for risk of bias. The methodological quality of studies included was assessed using the Mixed Methods Appraisal Tool (MMAT) [30]. A third reviewer was consulted when disagreements occurred. The MMAT can evaluate the primary studies' methodology of quantitative, qualitative, and mixed methods studies; therefore, it was deemed the most appropriate tool due to the variety of primary studies represented in the search return. The MMAT's validity and reliability have been documented previously as moderate to perfect regarding MMAT criteria and substantial concerning the overall quality score of appraised studies [31, 32]. The tool poses two questions for screening purposes and five questions about the methodological quality that differ for qualitative and quantitative study designs. There are three possible answers to each question ("yes," "no," or "can't tell"). For the five questions about methodological quality, every "yes" is converted to a score of 1 for a total summed score ranging from 0 to 5 [30].

### Data Synthesis

The final 46 papers were read multiple times by the first author to capture the focus of the investigation, the method, findings and implications of each study [33]. Following this, a thematic analysis was completed to identify consistent themes within the data [34]. Inductive analysis was used to determine the characteristics and features of sports school programmes. Deductive analysis was used to evaluate sports school impacts [33, 35] based on the Holistic Athletic Career model [14]. Deductive analysis focused on the four main themes of Wylleman’s [14] model: academic/vocational, athletic/physical, psychosocial and psychological impacts. Inductive analysis was then completed for the subthemes under each of the main four themes. In career development research, the Holistic Athletic Career model [14] has been recognised as one of the most comprehensive frameworks (see, e.g., [36]), describing an athlete’s career across multiple developmental dimensions (i.e., academic/vocational, athletic/physical, psychosocial and psychological). This model has been extensively used in research studies within sport to guide data collection about the athlete as a whole person (e.g., [37]). As such, this is why the current study used the Holistic Athletic Career model as a template for its thematic analysis. In this study, psychosocial impacts referred to impacts caused by the environment on the student-athlete’s social aspects. Psychological impacts referred to impacts related to the mental and emotional state of the student-athletes. There was a mix of quantitative and qualitative data across the studies; therefore, to find a "middle ground" [32], a narrative approach to synthesis was adopted to integrate, interpret and communicate the relevant finding [38, 39].

## Results

### Overview of Study Methodology

The 46 studies were conducted in Australia (*n* = 1), Belgium (*n* = 2), Bulgaria (*n* = 1), China (*n* = 1), Czech Republic (*n* = 1), Denmark (*n* = 4), Finland (*n* = 12), Germany (*n* = 6), Malaysia (*n* = 1), the Netherlands (*n* = 1), New Zealand (*n* = 2), Norway (*n* = 8), Singapore (*n* = 1), Slovenia (*n* = 1), Spain (*n* = 2), Sweden (*n* = 8) and the UK (*n* = 1). Sample size could only be determined based on the information provided in 45 studies, resulting in a total population of 11,036 sports school student-athletes, made up of 3,034 females, 3,746 males and 4,256 participants whose sex was not declared. Overall, 11 studies evaluated the characteristics and features of sports schools and 44 evaluated impacts across the four holistic athlete development themes (i.e., academic/vocational, *n* = 12; athletic/physical, *n* = 27; psychosocial, *n* = 9; psychological, *n* = 21). Of the 44 studies that evaluated impacts across holistic athlete development, only 11 studies measured across two of the holistic impact themes, seven studies measured across three themes, and no study measured across all four themes. The research designs used across the 46 studies included six quantitative descriptive, 25 quantitative non-randomised, 11 qualitative, and four mixed methods studies. A large number of data collection methods/instruments were used within the literature to evaluate the holistic athlete development impacts, including interviews (*n* = 15), non-specified questionnaires (*n* = 12), standardised questionnaires (*n* = 19), data from documents/materials (*n* = 6), field notes/observation (*n* = 6), clinical analysis (*n* = 1), physical and physiological assessments/analysis (*n* = 3), cross-case analysis (*n* = 1), researcher discussions (*n* = 1) and visual representations (*n* = 2). Table 2 presents the details of the 46 studies, including study design, study population and characteristics (i.e., sex, age, sport, type of school), the data collection method and instrument, and the key findings presented in accordance with the thematic analysis.

**Table 2:** Summary of study characteristics and findings for studies exploring the impacts of sport schools on holistic athlete development

Authors and year	Participant information	Method	Results and key findings	Thematic code
Andersson and Barker-Ruchti., 2018 [40]	7 female soccer players (at least 22 years old), who had been playing for the Swedish premier league for at least 3 years, attended a soccer high school and had been selected for the Swedish senior national soccer team	Semi-structured interviews and biographical mapping	Players struggled to manage the increasing school and soccer demands and felt that they were physically ill-prepared. The increasing school and soccer demands intensified their focus for soccer, but also resulted in a number of injuries. Upon leaving school, the players had not developed equally in soccer and education, but rather, prioritized soccer over education and thus did not continue their education	1, 2, 3a, 3b and 3d
Aunola et al. 2018 [46]	391 first year student-athletes (51% females and 49% males, mean age = 16, SD = 0.17) from six different upper secondary sport schools in Finland. A total of 50% of them represented individual sports and 50% team sports. Twenty percent of the athletes participated in Winter Olympic sports (e.g., alpine skiing, cross country skiing, ice hockey), 52% in Summer Olympic sports (e.g., athletics, football, swimming), and 28% in non-Olympic sports (e.g., orienteering, floorball, Finnish baseball)	Self-report questionnaire, exploring: task values for school work, task values for sport, educational aspirations, athletic career aspirations, type of sport, level of sport competition and grade point average (GPA)	The participants' GPA was, on average, 8.85 (SD = 0.62) at Time 1; 8.24 (SD = 0.88) at Time 2; and 8.05 (SD = 0.92) at Time 3. The dual-motivated pattern (characterized by high value placed on both school and sport) was most typical. However, the percentage of athletes demonstrating this pattern decreased over time, and the percentage showing a low academically motivated pattern increased	2, 3a and 3d
Baron-Thiene and Alfermann, 2015 [50]	125 (73 males, 52 females, mean age 16.2, SD = 0.65) students from five sport schools in Saxony. 69 (55%) participated in individual sports such as track and field, swimming, and diving in the summer and cross-country skiing, biathlon, and ice skating in the winter. The remaining 56 (45%) student-athletes participated in team sports such as basketball, handball, soccer, and volleyball	Demographic and sport-related data. A standardised questionnaire, the Sport Orientation Questionnaire (Elbe et al. 2009; German version) and the Volitional Components in Sport Questionnaire (Wenhold, Elbe, and Beckmann., 2009)	In the study, 29.6% of the student-athletes who participated at Time 1 had terminated their sport careers prematurely—a year later at Time 2, but were still pursuing their academic education. Dropouts scored significantly higher compared to-non-dropouts on the physical complaints’ subscales	2 and 3b
Boyadjieva and Steinhausen, 1996 [51]	Three nonclinical samples were studied:(a) special secondary school students (*n* = 91, mean age = 15.2, SD = 1.3); (b) standard secondary school students (* n* = 70, mean age = 14.7, SD = 0.9); and sport school students (* n* = 51, mean age = 16.2, SD = 1.3). The clinical sample comprised the entire cohort of consecutive admissions of 22 anorectic patients(20 females and 2 males, mean age = 15, SD = 2.0)	The Eating Attitude Test (EAT) (Garner, 1979) and the Eating Disorders Inventory (EDI) (Garner, 1991)	22 (10.4%) participants scored above the cut-off score of 30 on the EAT. Special school students dominated with 14 (15.4%) of the students, followed by 6 (8.6%) standard secondary school students and only 2 (3.9%) sport school students. In general, a similar picture emerged for the EDI	2 and 3b
Brand et al. 2013 [68]	866 elite student-athletes from a variety of sports (e.g. artistic gymnastics, boxing, canoe/kayak, cycling, handball, judo, modern pentathlon, rowing, shooting, soccer, swimming, track and field athletics, triathlon, volleyball, weight- lifting and wrestling), aged 12–15 years, enrolled in high-performance sport programming in German Elite Schools of Sport, 80 student-athletes from the same schools who have just been deselected from elite sport promotion, and 432 age- and sex-matched non-sport students from regular schools. Distributions of male and female students did not differ between the three study groups	Multidimensional Mood Questionnaire (Steyer et al. 1997) and an expanded 18-item version of the Composite International Diagnostic Screener (CID-S; Wittchen et al. 1999)	For female athletes, a number of symptoms (panic, posttraumatic stress, and specific phobia) were significantly less prevalent than in non-athletes. However, somatization was significantly more frequent. For males, the differences between samples were less pronounced. Deselected student-athletes exhibited lower mood scores (i.e., less positive chronic mood) compared to elite student-athletes as well as to non-athletes	2 and 3d
Brettschneider., 1999 [47]	711 male and female student-athletes from elite sport schools, aged between 12 and 17 years, who were competitors in various sports and 977 appropriately matched control group from regular schools. Overall, 822 males and 866 females	Data on timetables and training schedules. A modified version of the Self- Description Questionnaire (SDQ II) (Marsh, 1988, 1990) and narrative interviews	The majority of young athletes had few problems with school; the group has high academic achievement, which gives it a stable basis for developing self-confidence and self-esteem. Regarding the general self, adolescent athletes score significantly more positively than non-athletes, reflected in higher self-ratings in the social domain	2, 3a and 3c
Brown., 2014 [20]	20 elite athletes (age 14–18 years) and five teachers/coaches from two elite athlete programmes (EAPs), a state school with a sport academy option (School A) and a private correspondence school designed specifically for elite athletes (School B)	Semi-structured interviews, field notes during class visits and documents collected	Classifying students as high achievers, elite, motivated, strong, competitive and as ‘the really good people’ and distributing them into EAPs perpetuated an elitist discourse in both School A and School B that positioned elite athletes as having status, popularity and recognition, but it also created a source of frustration for those receiving little recognition within the EAP. Furthermore, the elite athletes and sponsors promoted the EAPs and in turn the EAPs and sponsors promoted the achievements and successes of the elite athletes as their skills and knowledge were highly valued in comparison to other students within the school. However, the EAPs offered limited post-school options of obtaining an athletic scholarship to study at a university and/or to become a professional athlete	2, 3b and 3c
Brown., 2016 [27]	20 elite athletes (age 14–18 years) and five teachers/coaches from two EAPs, a state school with a sport academy option (School A) and a private correspondence school designed specifically for elite athletes (School B)	Field notes and photos during school visits, information from school websites and interviews with the teachers/coaches (individually) and elite athletes using semi-structured interviews and two focus group interviews	The EAPs emphasised corporate values of loyalty, self-sacrifice and work ethic and perpetuated the dichotomies of theory/practice, thinking/doing and mind/body discourses that assisted in the marginalised academic status of the EAP. Most of the elite athletes struggled to reconcile their athletic identity with their teenage identity as they sacrificed time with friends, pleasures such as frozen colas and other pursuits to be role models for younger athletes and others in their community	1, 2, 3a, 3b and 3c
Chua., 2015 [65]	13 participants – dance students (* n* = 4), teachers (* n* = 6), parents (* n* = 2), and one sibling. Students aged 16 to 22 years were enrolled in their national dance institutions— the Finnish National Opera Ballet School and the Singapore Dance Theatre	Data were documents, letters, interviews, and observation field notes collected over 2 years	Peers were important sources of emotional and informational support. The Finnish students spend a great deal of time together in class, rehearsals, and leisure throughout the school term, pursuing a common career goal that probably spurred them to support one another. Conversely, missing from the data was the influence of friends in the Singaporean students’ talent development. Vicarious experience or observing a peer succeed at a task strengthened self-efficacy in ballet	2 and 3c
De Bosscher et al. 2016 [41]	408 athletes within an Elite Sport School (ESS) (188 males, 220 females, < 18 years = 10.5%, 18–23 years = 66.5%, 24–28 years = 23%) and 341 from athletes outside ESS (50% male, 50% female, < 18 years = 8.4%, 18–23 years = 51.3%, 24–28 years = 40.3%). 253 athletes from team sports and 496 individual sport athletes	Data from Bloso (Flemish sports agency), lengthy surveys and 10 semi-structured interviews	The data showed no clear evidence of more effective outputs (performance), or more positive evaluation of throughputs (processes) by athletes who attended an ESS. Athletes who did not attend an ESS received less support services, but those who did receive such services were generally more satisfied. They were equally satisfied about their coaches’ expertise. 95% of all students within an ESS attained their diploma in secondary education. No significant differences between elite athletes within and outside ESS on continuation to higher education after secondary school	1, 2, 3a and 3b
Elbe et al. 2005 [69]	327 students attending a school for young elite athletes (157 males and 170 females) of whom 74 lived in the on-campus boarding school. The age groups are divided according to classes, with 12- to 13-year-olds in grades seven to eight (*n* = 98), 14- to 15-year-olds in grades nine to 10 (*n* = 138), 16- to 17-year-olds in grades 11 and 12 (*n* = 61) and the 18-year-olds in grade 13 (*n* = 30)	Volitional Components Questionnaire (Kuhl and Fuhrmann, 1998)	Young elite athletes in comparison with students of a regular school show higher values in self-optimisation and stayed at this higher level during the course of the study. A comparison concerning the living situation shows a more positive development in self-optimisation for those athletes living on campus	2 and 3d
Emrich et al. 2009 [22]	196 German participants (32 consistently in ESS, 39 partly in ESS, 125 from never in ESS) participants of the 2004 Summer Olympic Games as well as the 2006 Winter Olympic Games. No age difference between the individual categories	A standardized survey	There was no difference in athletic performances (medals won) between ESS pupils and others in the 2004 Summer Olympics, while in the 2006 Winter Olympics, there was a significant difference (substantially higher share of medals amount ESS pupils than for pupils who did not attend an ESS). Furthermore, there were no differences in school performances between the groups. Missed examinations owing to competitions and missed lessons due to competitions were scenarios often experienced by sport school student-athletes. Pupils at ESS often go on to pursue careers in the federal police and the armed forces, while many more non-ESS pupils work toward earning a university degree	2, 3a, 3b and 3d
Eriksson et al. 2017 [52]	244 skiers at the Swedish National Elite Sport Schools for cross-country skiing, biathlon, and ski-orienteering (127 males and 117 females, mean age 16.8, SD = 1.2) and 238 adolescents (109 males and 127 females, mean age = 17.6, SD = 1.1) reference group, matched for sport school municipalities	Postal questionnaires	The proportion of participants with self-reported physician-diagnosed asthma was higher among skiers than in the reference group. The median age at asthma onset was higher among skiers than in the reference group. Female sex, family history of asthma, nasal allergy, and being a skier were risk factors associated with self-reported physician-diagnosed asthma	2 and 3b
Gisslèn et al. 2005 [53]	57 students at the Swedish National Centre for high school volleyball (29 males and 28 females, mean age = 17.4) and 55 (27 males and 38 females, mean age = 17.4) non-regularly sports active controls	The patellar tendons were evaluated clinically and by grey scale ultrasonography and power Doppler sonography	A clinical diagnosis of jumper’s knee, together with structural tendon changes and neovascularisation visualised with sonography, was seen among Swedish elite junior volleyball players but not in matched not regularly sports active controls	2 and 3b
Henriksen et al. 2011 [42]	Athletes who attend Wang School of Elite Sports kayak program (age 16–19 years) and elite athletes, coaches, managers and parents from the environment	Data from interviews, participant observation and document analysis, as described more fully in Henriksen (2010)	All coaches are former elite athletes raised within the system. One main feature is the relationship between prospects and a community of more elite athletes, which was at the heart of the environment. The elite athletes were really visible as role models, and arguably training with the elite level athletes may prepare the prospects for the next phase in their athletic career and so ease their transition. A second such feature relates to the athletes’ experience of living in an integrated and coordinated environment. The kayakers experienced an integrated set of “pulls”, which they attributed to a good coordination and communication among different components in the environment. Final feature is the way in which the environment allowed space for the athletes to have other personal identities than their athletic one (e.g., a student, a friend, a mentor of younger athletes) and encouraged them to develop qualities and skills applicable not only in sport but also in other spheres of life	1, 2, 3b, 3c and 3d
Ingrell et al. 2019 [70]	78 student-athletes (30 female and 48 males, mean age at T1 = 12.7 years, SD = 0.44), attending a sport compulsory school. The sports represented by the participants in this cohort were soccer, ice hockey, figure skating, floorball, swimming, diving, basketball, badminton, and tennis	Swedish version of Athlete Burnout Questionnaire (Raedeke and Smith, 2011, 2009) and Swedish and version of the Task and Ego Orientation in Sport Questionnaire (Duda and Nicholls, 1992)	Increases in all three (reduced sense of accomplishment, emotional and physical exhaustion, and sports devaluation) burnout variables, therefore burnout scores increased over the three-year period. Furthermore, task orientation had a negative within-person effect on burnout perceptions with regard to a reduced sense of accomplishment and sport devaluation among student-athletes	2 and 3d
Into et al. 2020 [71]	414 student-athletes (age 17–18 years, 49% female, 51% male), from seven sports high schools participated in this study. In the sample, 47.3% and 52.4% of the adolescents participated in individual and team sports, respectively	School Burnout Inventory (Salmela-Aro, Kiuru, et al. 2009), Sport Burnout Inventory—Dual Career (Sorkkila et al. 20,172,017) and the Empowering and Disempowering Motivational Climate Questionnaire (Appleton et al. 2016)	4 groups of experienced coaching climates were identified: extremely disempowering (7%), disempowering (27%), empowering (24%), and intermediate (42%). Overall, student-athletes in the extremely disempowering and disempowering coaching climate groups reported higher levels of sport and school burnout than student-athletes in the other 2 groups	2 and 3d
Knowles et al. 2017 [23]	233 students (74% male and 26% female, mean age = 14.3, SD = 1.6); 187 student-athletes and 46 non-sport school students from one large metropolitan school in Australia. Student-athletes participated in the sport for which they were selected into the school; basketball (24%), netball (8%), football (AFL, 31%) or soccer (35%)	Online survey that captured information about time use, sport involvement and health and wellbeing	Sport school students spent less time in sedentary leisure and similar time studying to non-sport school students and had better general health and social and emotional wellbeing than non-sport school students. Student-athlete burnout scores for reduced sense of accomplishment, emotional and physical exhaustion and devaluation of sport all indicated relatively low levels of burnout	2, 3b and 3d
Kristiansen and Houlihan., 2015 [24]	35 respondents from nine stakeholder groups, including athletes (*n* = 7, from summer and winter sports), coaches (*n* = 7), teachers (*n* = 4), elite entourage members (*n* = 3), parents (*n* = 8), sport school managers (*n* = 5), Olympiatoppen centre (*n* = 4) and federations (*n* = 4)	Data were collected through a series of interviews. The interview guide was tailored to the different participants and their stakeholder position	The quality of coaches working at the Norwegian College of Elite Sport (NTG) is considered to be a significant marketing advantage. The resources available at NTG enable athletes to be given extra tutoring ‘to help after longer period of absence,’ add extra hours (of tuition)' to keep up with school and if students are away at training camps or at competitions as well as having access to the services of nutritionists, nurses, physiotherapists and other support personnel to deal with issues related to their athletic career. Having these resources ‘in-house’, is an advantage that was mentioned by both the athletes and parents	1
Kristiansen., 2018 [28]	26 Norwegian athletes who qualified for the European Youth Olympic Festival (11 females and 15 males, mean age = 16.65 years, SD = 0.91). Athletes competed in cross-country skiing, biathlon, alpine skiing, ski jumping, figure skating and Nordic skiing, respectively. Overall, 19 of the athletes attended a private sport school, 10 athletes a sports programme at public schools and 4 were still in lower secondary school	Mixed methods survey and the author observed three pre-camps hosted by Olympiatoppen. Observations were also made during the competition	Pursuing a dual career is often a challenging balancing act for the young student-athletes. Additional results identified the importance of supportive parents, schools that adapt the workload for the student-athletes, and a federation that recognizes the important role of parents and schools	1, 2 and 3d
Lichtenstein et al. 2018 [54]	Three high-risk samples (*n* = 471): 257 sport school students (mean age = 15.8, SD = 1.25), 127 fitness centre attendees (mean age = 17.6 years, SD = 1.41), and 87 patients with eating disorder diagnoses (mean age = 15.8, SD = 2.33)	A survey which included the youth version of the Exercise Addiction Inventory (Griffiths et al. 2005), the SCOFF Questionnaire for eating disorders, sociodemographic items, and questions concerning disturbed attitudes toward exercise and eating	The prevalence rate of exercise addiction was 4.0% in sport school athletes, 8.7% in fitness attendees, and 21% in patients with eating disorders. Exercise addiction was associated with feelings of guilt when not exercising, ignoring pain and injury, and higher levels of body dissatisfaction	2 and 3b
Martinsen and Sundgot-Borgen., 2013 [55]	306 elite athletes attending Elite Sport High Schools in Norway (204 males and 102 females, mean age = 16.5 years, SD = 0.3) and 244 controls from two randomly selected regular high schools in Norway (79 males and 100 females, mean age = 16.9, SD = 0.3), representing 50 different sports/disciplines	This was a two-phase study, including a self-report questionnaire (part I) followed by clinical interviews (part II)	In part I, more controls than athletes were classified as ‘‘at risk’’ for eating disorders (ED). In part II, the prevalence of ED among the total population of athletes and controls was estimated to be 7.0% versus 2.3%, with a difference of 4.7%, with the ED prevalence being higher for female than male athletes and female and male controls. No difference in the prevalence of ED was detected between the females in weight-sensitive and less weight-sensitive sport groups	2 and 3b
Martinsen et al. 2010 [56]	First-year students (15–16 years old) of 16 different Norwegian Elite Sport High Schools (*n* = 682), two randomly selected ordinary high schools from a county representative of the general Norwegian population (*n* = 423) and a birth date in 1992 (i.e., age of 15 or 16 at the time of data collection). The athletes represented 50 different sports	Questionnaire and Eating Disorders Inventory –2 (Garner, 1991)	A higher percentage of controls than athletes reported dieting and use of pathogenic weight-control methods. The most frequent reason for dieting among girl and boy controls and girl athletes was to improve appearance, whereas boy athletes most often reported enhanced performance as a reason for dieting. One-third of the athlete boys and 13% of the athlete girls were dieting as directed by their coach or teacher, and this was higher than among boy and girl controls respectively	2 and 3b
Moazami-Goodarzi et al. 2020 [72]	Student-athletes from six Finnish upper secondary sport schools (*N* = 391, mean age at T1 = 16, SD = 0.17; 51% females and 49% males). 50% played individual sports (e.g., swimming or athletics) and 50% team sports (e.g., ice hockey or football)	Sports achievement 4-point Likert scale, grade point average, the Athletic Identity Measurement Scale (Brewer et al. 1993) and modified Athletic Identity Measurement Scale (Brewer et al. 1993)	Three groups were identified: dual identity (77%), changing identity (5%), and athletic identity (18%). The higher the academic achievement was at Time 1, the more likely the athletes were to show a dual identity than an athletic identity profile. Similarly, athletes with dual identity showed higher subsequent academic achievement at Time 4 than those with an athletic identity profile. Finally, athletes with dual identity were more likely and athletes with athletic identity less likely to withdraw from sport activities during upper secondary school than would be expected by chance	2 and 3d
Morris et al. 2020 [19]	Interview participants (*N* = 31) were purposefully selected because they held an understanding of one or more dual career development environments (DCDE). The expert discussion participants included practitioners (*N* = 4) and researchers (*N* = 13) who had significant experience within the area of dual careers, in some cases over 20 years’ experience	Documentary analysis, interviews with knowledgeable stakeholders, cross-case analysis, and researcher discussions	They are situated in upper and lower general and vocational secondary education (ISCED level 2–5). Data highlight that the majority of programs support athletes through development and mastery phases of their athletic development. Sport schools can either be a) education-led or vocation-led system (i.e., the athlete is based in an education/vocation environment which offers support for sport and performance, or (b) a combined dual career development environment (i.e., an organization or institution that works in tandem with both sport and education/vocational providers to deliver an all-round support package to the individual undertaking the dual career). The support provisions between institutions in the same country are not standardized because each is able to decide the provision of support, they give to each athlete for themselves – they can, however, include similar features (e.g., sports facilities, academic support and sport science provision)	1
Moseid et al. 2019a [57]	259 elite athletes (16-year-olds, 68% male and 32% female) from three specialised Sport Academy High Schools in Norway. Thirty different sport disciplines (both summer and winter sports from both individual and team sports) were represented and grouped into three major categories (endurance [*n* = 69], technical [*n* = 62], and team sports [*n* = 128])	Web‐based questionnaire and the Oslo Sports Trauma Research Centre (Clarsen et all., 2014) questionnaire on health problems	In this specialized Sport Academy High School program, 39% of the athletes reported early specialization (at 12 years or younger). However, early specialization did not increase the risk of injury or illness during the 26 weeks, nor did being a single‐sport athlete the previous two years increase this risk. The best performing athletes at the time of enrolment were not at greater risk of becoming injured or ill during the 26 weeks	2 and 3b
Moseid et al. 2019b [58]	166 Sport Academy High School youth elite athletes (age 15–16 years, 72% males and 28% female) from a variety of team, technical, and endurance sports newly enrolled into specialized sport academy high schools	The Oslo Sports Trauma Research Center Questionnaire (Clarsen et all., 2014) on Health Problems and Ironman Jr physical fitness test battery	During the 26‐week period, the athletes reported 156 overuse injuries, 146 acute injuries, and 294 illnesses. Each athlete reported an average of 3.6 health problems. Overall, the least fit quartile of athletes did not report more health problems compared with the rest of the cohort	2 and 3b
Mudrak and Zabrodska., 2014 [43]	Nine young gifted children (five female and four males, aged between 17 and 23 years). Three of the participants (1 female, 2 male) attended sport schools and achieved, in childhood, an extraordinary level in sport, specifically gymnastics and taekwondo	Semi-structured interviews	Both sport school athletes described gradually losing a sense of agency in their future development. They described situations in which their excellent early results led to increasing expectations and pressure to successfully compete with other children. They both experienced a significant decrease in their originally very high motivation and increase feelings of psychological and physical burnout and quit competitive sport altogether. Because of their intensive engagement in sport practice, they had had only limited experience with “ordinary” life outside competitive sport. After withdrawing from competitive sport, they experienced only a very limited sense of agency in relation to other possible professional careers and had difficulties in finding a new direction in life	1, 2, 3a and 3d
Perez-Rivases et al. 2020 [48]	72 Spanish female student-athletes (mean age = 17.33 years; SD = 0.73), who were grant holders in talent development centres or high-performance centres and studying upper secondary school. Participants took part in both individual (47.2%) and team (52.8%) sports	Spanish versions of the Dual Career Competency Questionnaire for Athletes (De Brandt et al. 2018) and Dual Career Competency Questionnaire for Athletes with scenario extension (De Brandt, 2017)	Participants perceived the need to better develop all their dual career (DC) competencies (e.g., “ability to resolve conflicts”; “ability to use your time efficiently”). Results show that trying to combine social life with DC (92.4%), missing significant days of study (86.6%), and having a challenging study year (79.4%) were the three scenarios most experienced by female student-athletes. Similarly, suffering from an injury was reported as experienced by 46 (69.7%) of the participants	2, 3a and 3b
Rasyid et al. 2020 [73]	854 young athletes from two Malaysian Sport Schools (age 13–18 years old)	Modified version of the School Burnout Inventory (Salmela-Aro and Naatanen, 2005), School Burnout Inventory (Salmela-Aro and Naatanen, 2005), Success Expectations Scale (Nurmi, Salmela-Aro, and Haavisto, 1995), modified version of the parental belief’s questionnaire	Athletes were more inclined toward Task orientation. Male were more task and ego orientated than females. Younger athletes are more task-oriented as compared to senior athletes. Individual sport athletes were found to be more Ego oriented than team sport athletes	2 and 3d
Romar., 2012 [44]	49 students (15 females and 34 males, mean age = 17 years) from three skiing boarding schools, two cross-country and one alpine school	Questionnaire about academic success and athletic performance	The results showed that 80% of the students extended their high school studies from three to four years. Fifty-four percent of alpine skiers and 15% of cross-country skiers indicated that their best athletic success was in international competitions. Finnish alpine and cross-country athletes missed on average 88 and 22 of 190 days per academic year. Almost all students perceived that skiing school helped by combining sport and school. However, only 40% of the alpine skiers and 62% of the cross-country skiers were satisfied with their present athletic success. Seventy-three percent of the alpine skiers felt that sport participation affected negatively their success in school. Success in sport, good training possibilities, skilled coaches and caring friends were reason for enjoying life in skiing boarding schools	1, 2, 3a and 3b
Ronkainen et al. 2020 [59]	17 international level Finnish student-athletes pursuing sport and education in upper secondary sport schools (7 males and 10 females, age 16–17 years). Eleven athletes participated in individual sports (judo, tennis, athletics, swimming, artistic gymnastics, alpine skiing, ski orienteering, and cross-country skiing) and six athletes participated in team sports (football, ice hockey, basketball, and artistic group gymnastics)	Visual representations of their “dream days” and low-structured interviews where participants were invited to tell a story about the best possible day sometime in the future	They identified three types of dream days: a day on holiday, focused on relaxation, having a good time with friends, and recreational activities; a day of peak athletic performance describing winning a major competition; and a regular day involving school or work, athletic training and time with family. They concluded that the short future timespan and a low number of sporting dream days might indicate overload and lack of time for reflection	2 and 3c
Ronkainen and Ryba., 2018 [66]	10 female Finnish youth athletes participating in the national talent development programme and studying in upper-secondary sport schools (age at baseline: 15–16 years)	In depth interviews	Summarised an account of three athletes. One athlete was on track with her life plan, had graduated with excellent grades and received an athletic scholarship to the USA and sustained a dual-career throughout upper secondary school and into higher education. Two of the athletes equipped themselves with 'skills' to manage and organise time. One of the athletes indicated that she needs to achieve perfection every day in order to feel good about herself finding it difficult to be satisfied with normal performance. This led to excessive training regimes and subsequent injury. The final athletes felt that she did not have time, that sport was stealing time from her schoolwork and from being with friends. She experienced symptoms of burnout, both in sport and school	2, 3b, 3c, and 3d
Rosendahl et al. 2009 [60]	576 young athletes of Elite Sports Schools in Germany (210 females and 366 males, mean age = 15.7 years, SD = 1.25) and a reference group consisting of 291 non-athletes from regular high schools (169 females and 122 males, mean age = 15.9 years, SD = 0.90). The athletes competed in 26 different sports representing technical, endurance, esthetic, weight class, ball game, power and antigravitation sports	Eating Attitude Test (Garner et al. 1982; German version). Body image and body ideal were measured with male and female silhouettes representing different weight categories. The body mass index (BMI) was calculated as weight in kilograms divided by the square of the height in meters (kg/ m2)	Athletes did not show a higher frequency of disordered eating than non-athletes. Gender and dietary experience, but not group (athletes vs non-athletes), were significant predictors of disordered eating. It can be concluded that dietary experience and female gender proved to be important risk factors of disordered eating. Participation in sports seems to be protective for developing serious eating problems, especially in girls	2 and 3b
Ryba et al. 2017 [36]	18 (10 females and 8 males) elite junior athletes, aged 15–16 years at baseline, identified through the Finnish Sport Academies under the auspices of National Olympic Committee	Two individual conversational interviews	Thirteen of 18 adolescent athletes drew primarily on the performance narrative plot to construct their life story and five of 18 athletes could not project into the future beyond their athletic selves. Constructing their identities using the narrative resources of the performance plot, young athletes’ stories revolved around winning or being the best, training hard, competing and achieving in the senior ranks. While at the time of this research, all 18 participants were integrating sport and education in their daily living, most of the adolescents considered school activities to be the inevitable part of youth, which consumed all their “free” time after sport, and five of them had difficulties to imagine themselves to be anything but professional athletes in the future	2, 3a and 3b
Sandström et al. 2012 [61]	57 female athletes at a senior high school for top-level athletes (mean age = 16.8 years, SD = 0.9). The control group consisted of 92 (mean age = 17.1 years, SD = 0.9) non-athlete students. The athletes practiced different sports, both individual and team	Questionnaires and levels of haemoglobin, serum iron, total iron-binding capacity, transferrin saturation, and serum ferritin	The main result of the study is the finding that iron deficiency (ID) and iron deficiency anaemia (IDA) are common among young adolescent female athletes and that there was no difference between female athletes and nonathletes. Athletes reported a significantly higher consumption of milk a day, ate more often and were smokers to a less extent compared with nonathletes	2 and 3b
Skrubbeltrang et al. 2020 [45]	All Sports Class students in 7th–9th grades (age 13–16 years, * n* = 1170, 733 males and 437 females) in schools in 15 Team Denmark-supported municipalities. More than half the athletes played football and handball. After this, swimming, ice hockey, badminton and basketball follow in relative popularity	Survey of the student population in 2013 and a follow-up sample in 2015	Three-quarters of the Sports Class students agreed that the classes provided better opportunities. 44% of boys compared to 33% of girls indicated that the morning practices helped them improve “to a great extent.” 51% stated at times they couldn't be bothered to invest the time and energy necessary to reach the elite. 49% said they had pushed themselves so much that it affected their enjoyment in their sport and 51% pushed themselves so much that they sustained injuries	1, 2, 3b and 3d
Skrubbeltrang et al. 2016 [67]	74 sport students (29 females and 45 males), grade 8 (age 14–15 years) and 12 (age 18–19 years) with some of the students in grade 9 (age 15–16 years), as well as parents, teachers and trainers from four schools located in four regions of Denmark	Participant observation of student/parents/teacher/club meetings, as well as classroom observations. In addition, 74 interviews with sport students (48 individual and 7 interviews in pairs) and trainers, teachers and some of the parents. Finally, a collaborative team ethnography	In the sports classes, they found that there is a code of conduct, whereby the sports students as a learning subject must commit to working hard to develop themselves as athletic talents – and they should also have the same attitude towards their schoolwork. Student-athletes have less time for peers outside of sport and must continually negotiate the terms of their membership of this group, for example, that they attend activities less frequently. They argue that sport schools oblige students to follow a narrow developmental track with an ambitious goal of performing in both sport and school, and that this is threatened when a sports student prioritises either sport or school while he/she is still enrolled in the class	2 and 3c
Sorkkila et al. 2017 [18]	391 student-athletes (51% females and 49% males, mean age = 16, SD = 0.17) from six different upper secondary sport schools in Finland, and 448 parents (58% mothers). Out of the participating student-athletes, 197 (50%) played individual sports (e.g., athletics or judo) and 194 (50%) played team sports (e.g., football or ice hockey)	Modified version of the School Burnout Inventory (Salmela-Aro and Naatanen, 2005), School Burnout Inventory (Salmela-Aro and Naatanen, 2005), Success Expectations Scale (Nurmi, Salmela-Aro, and Haavisto, 1995), modified version of the parental belief’s questionnaires (Nurmi et al. 1995)	Four burnout profiles were identified: well-functioning (60%), mild sport burnout (28%), school burnout (9.6%), and severe sport burnout (2.7%). Athletes' and parents' expectations of success seemed to protect against burnout in the same domain, but this protection did not extend to the other domain. Moreover, high success expectations in one domain seemed to increase the risk for burnout in another domain	2 and 3d
Sorkkila et al. 2018 [74]	391 Finnish student-athletes (51% females and 49% males) from six upper secondary sport schools, age 15–16 years (mean age = 16, SD = 0.17). Fifty percent of the participants practiced individual sports and 50% team sports	School Burnout Inventory (Salmela-Aro, Kiuru, et al. 2009) and modified Perception of Success Questionnaire (Roberts et al. 1998)	Burnout dimensions in a particular domain were substantially stable within the same domain during the first year of upper secondary school and that school-related exhaustion at the beginning of upper secondary school predicted sport-related exhaustion at the end of the school year. Mastery goals in sport and school were negatively associated with cynicism and feelings of inadequacy within the same domain. Furthermore, performance goals in school were positively associated with school-related cynicism	2 and 3d
Sorkkila et al. 2019 [75]	391 first year student‐athletes (51% females and 49% males, mean age = 16, SD = 0.17) from six different upper secondary sport schools in Finland. A total of 50% of them represented individual sports and 50% team sports	Sport Burnout Inventory—Dual Career Form (Sorkkila et al. 2017), School Burnout Inventory (Salmela-Aro, Kiuru, et al. 2009) and Brief Resilience Scale (Fletcher and Sakar, 2013)	Three burnout profiles were identified: (a) The Average profile (60%) (b) The Increased burnout profile (32%), and (c) the Non‐risk profile (8%). Increased burnout group symptoms were less resilient and more likely to dropout from sport than those in the other two groups. Furthermore, those in the Non‐risk profile were more resilient than athletes in the other two groups	2 and 3d
Stambulova et al. 2015 [37]	16-year-old male and female student-athletes, representative of 27 different individual (e.g., track-and-field, tennis, cycling, golf) and team (e.g., basketball, handball, hockey) sports and 33 national elite sport schools across Sweden (*n* = 261 in the first and * n* = 250 in the second measurement)	The Dual Career Survey (Engstrom and Stambulova, 2011a), The Athletic Identity Measurement Scale (Brewer, Van Raalte, and Linder, 1993), and The Student Identity Measurement Scale (Engstrom and Stambulova, 2011b) and in-depth interviews	Student-athletes' adaptation at RIGs was related to coordinating different layers of their development (athletic, psychological, psychosocial, and educational) in order to search for, and (possibly) obtain an optimal balance between sport, studies and private life. The participants of the study perceived all the three big spheres of their life examined in the study (sport, studies and private life) as important and demanding, both at the beginning and at the end of their first educational year at RIG, and used resources and coping efforts to deal with them	1, 2, 3a, 3c and 3d
Stenling et al. 2015 [62]	A total of 247 elite skiers (109 females, 138 males) athletes from 18 sport high schools in Sweden. The athletes’ age ranged from 16 to 20 years (mean age = 17.8 years; *SD* = 0.9)	Questionnaires assessing perceived autonomy support from the coach, need satisfaction, motivation, and psychological well-being	Initial level of need satisfaction at Time 1 negatively predicted change in perceived autonomy support, motivation, and well-being, and initial level of motivation at Time 1 positively predicted change in perceived autonomy support and change in well-being. Correlations between intraindividual changes were all positively correlated and the athletes reported high and stable levels of perceived autonomy support, need satisfaction, self-determination index, and well-being over the course of the competitive season	2 and 3b
Stornæs et al. 2019 [63]	832 adolescents (13–14 years old, 53% females and 47% males): 166 students (82 females and 84 males) from elite sports- /performance-oriented lower secondary schools, and 666 students from ordinary schools (361 females and 305 males)	Two perfectionism scales: the child-adolescent perfectionism scale (Flett et al. 2000) and the frost multidimensional perfectionism scale (Frost et al. 1990)	A higher relative proportion of ordinary school girls (39.3%) compared to elite school girls (25.6%), and ordinary school boys (36.4%) compared to elite school boys (19%), were observed within profile 1 and profile 2 (low self-oriented perfectionism with high perfectionistic concerns). Profile 1 and 2 were associated with the highest levels on anxiety, depression and excessive weight and shape concerns, and the lowest ratings for resilience and global self-worth	2, 3b and 3d
van Rens et al. 2012 [49]	242 (former) athletes who were labelled by their sport federations as talented athletes during the years 2004–2008 (46% male and 54% female, mean age = 21 years, SD = 2.8). 70% did not attend a Topsport Talent School (TTS). Tennis players and gymnasts were overrepresented at TTS, speed-skaters were overrepresented at mainstream secondary schools. The sports speed-skating (34%) and judo (18%) were most often represented in the sample	Online questionnaire based on: whether they attended a TTS, sport performance level, school performance level, commitment to sport during secondary school, and satisfaction with the combination of school and sport	Attending a TTS did not influence the current and highest attained sport performance levels of talented athletes (at both junior and senior level). Neither were talents who had attended a TTS more satisfied about the combination of school and sport, nor were they more motivated for their sport. Furthermore, results indicated that talents who had attended TTS were less motivated to do well in school; attained lower educational levels in both their secondary school and further education and were less likely to pursue higher education	2, 3a, 3b and 3d
Zhao et al. 2020 [64]	Male student-athletes (*N* = 21, mean age 12.14 ± 0.62 years) from the Shanghai Elite Sport school. Categorized into two groups: swimming group (10 athletes), and the racket sports group (11 players: 7 table tennis and 4 badminton players)	Physiological measurements (vital capacity (VC), haemoglobin (Hb) concentration, heart rate at rest), anthropometric parameters (body height, body weight, chest girth), and motor tests (back strength (BS), complex reaction speed)	Over the 2-year investigation Hb and VC linearly increase between the ages of 12 and 14 years, not only reflecting their sports-specific response to training, but also the impact of testosterone production during the onset of puberty. The resting HR remained on the same level. In the racket sports group, the dynamic BS increased over the two years by 44.0%. In the swimmers' group, the dynamic BS increased until a certain levelling of developed during the last half year	2 and 3b

Thematic code: (1) characteristics and features of sport school programmes; (2) methods used to evaluate sport school impacts and outcomes; (3a) academic/vocational impacts and outcomes associated with sport school programs; (3b) athletic/physical impacts and outcomes associated with sport school programs; (3c) psychosocial impacts and outcomes associated with sport school programs; and (3d) psychological impacts and outcomes associated with sport school programs

### Study Quality

The scores for the assessment of study quality according to MMAT [29] are presented in Table 3 with a description of the study quality criteria presented in Table 4. The study quality ranged from two to five out of the five items assessed with a mean score of 4.39 (SD = 0.95), with study quality for quantitative descriptive 4.00 (SD = 1.26), quantitative non-randomised 4.36 (SD = 0.99), qualitative 4.73 (SD = 0.65) and mixed methods 4.25 (SD = 0.96), respectively. No study was excluded based on methodological quality.

**Table 3:** Methodological quality scale assessment

Study	Country	Design	Methodological quality criteria					Total quality assessment score
			1	2	3	4	5	
Andersson and Barker-Ruchti 2018 [40]	Sweden	Mixed methods	1	1	1	1	1	5
Aunola et al. 2018 [46]	Finland	Quantitative non-randomised	1	1	1	1	1	5
Baron-Thiene and Alfermann, 2015 [50]	Germany	Quantitative non-randomised	1	1	1	1	1	5
Boyadjieva and Steinhausen, 1996 [51]	Bulgaria	Quantitative non-randomised	1	0	1	0	1	3
Brand et al. 2013 [68]	Germany	Quantitative non-randomised	1	1	1	1	1	5
Brettschneider 1999 [47]	Germany	Mixed methods	1	1	0	0	1	3
Brown 2014 [20]	New Zealand	Qualitative	1	1	1	1	1	5
Brown 2016 [27]	New Zealand	Qualitative	1	1	1	1	1	5
Chua 2015 [65]	Finland and Singapore	Qualitative	1	0	1	1	1	4
De Bosscher et al. 2016 [41]	Belgium	Quantitative non-randomised	1	1	0	1	0	3
Elbe et al. 2005 [69]	Germany	Quantitative non-randomised	1	1	1	1	1	5
Emrich et al. 2009 [22]	Germany	Quantitative non-randomised	1	0	0	0	1	2
Eriksson et al. 2017 [52]	Sweden	Quantitative non-randomised	1	1	1	1	1	5
Gisslèn et al. 2005 [53]	Sweden	Quantitative non-randomised	1	1	1	1	1	5
Henriksen et al. 2011 [42]	Denmark	Qualitative	1	1	1	1	1	5
Ingrell et al. 2019 [70]	Sweden	Quantitative non-randomised	1	1	0	0	1	3
Into et al. 2020 [71]	Finland	Quantitative non-randomised	1	1	1	1	1	5
Knowles et al. 2017 [23]	Australia	Quantitative non-randomised	1	1	1	1	1	5
Kristiansen and Houlihan 2015 [24]	Norway	Qualitative	1	1	1	1	1	5
Kristiansen 2018 [28]	Norway	Mixed methods	0	1	1	1	1	4
Lichtenstein et al. 2018 [54]	Denmark	Quantitative non-randomised	1	1	0	0	1	3
Martinsen and Sundgot-Borgen 2013 [55]	Norway	Quantitative non-randomised	1	1	1	1	1	5
Martinsen et al. 2010 [56]	Norway	Quantitative non-randomised	1	1	1	1	1	5
Moazami-Goodarzi et al. 2020 [72]	Finland	Quantitative non-randomised	1	1	0	1	0	3
Morris et al. 2020 [19]	Multi-Nation (Belgium, Denmark, Finland, Slovenia, Spain, Sweden, UK)	Qualitative	1	1	0	0	1	3
Moseid et al. 2019a [57]	Norway	Quantitative non-randomised	1	1	1	1	1	5
Moseid et al. 2019b [58]	Norway	Quantitative non-randomised	1	1	1	1	1	5
Mudrak and Zabrodska 2014 [43]	Czech Republic	Qualitative	1	1	1	1	1	5
Perez-Rivases et al. 2020 [48]	Spain	Quantitative non-randomised	1	1	1	1	1	5
Rasyid et al. 2020 [73]	Malaysia	Quantitative descriptive	1	1	1	1	1	5
Romar 2012 [44]	Finland	Quantitative descriptive	0	1	0	1	0	2
Ronkainen et al. 2020 [59]	Finland	Qualitative	1	1	1	1	1	5
Ronkainen and Ryba 2018 [66]	Finland	Qualitative	1	1	1	1	1	5
Rosendahl et al. 2009 [60]	Germany	Quantitative descriptive	1	1	1	1	1	5
Ryba et al. 2017 [36]	Finland	Qualitative	1	1	1	1	1	5
Sandström et al. 2012 [61]	Sweden	Quantitative non-randomised	1	1	1	1	1	5
Skrubbeltrang et al. 2020 [45]	Denmark	Quantitative descriptive	1	1	0	1	0	3
Skrubbeltrang et al. 2016 [67]	Denmark	Qualitative	1	1	1	1	1	5
Sorkkila et al. 2017 [18]	Finland	Quantitative descriptive	1	1	1	1	1	5
Sorkkila et al. 2018 [74]	Finland	Quantitative non-randomised	1	1	1	1	1	5
Sorkkila et al. 2019 [75]	Sweden	Quantitative non-randomised	1	1	1	1	1	5
Stambulova et al. 2015 [37]	Sweden	Mixed methods	1	1	1	1	1	5
Stenling et al. 2015 [62]	Sweden	Quantitative descriptive	1	1	1	0	1	4
Stornæs et al. 2019 [63]	Norway	Quantitative non-randomised	1	1	1	1	1	5
van Rens et al. 2012 [49]	Netherlands	Quantitative non-randomised	1	0	1	1	0	3
Zhao et al. 2020 [64]	China	Quantitative non-randomised	1	1	1	0	1	4

**Table 4:** Descriptor of study quality criteria

	Mixed methods	Qualitative	Quantitative descriptive	Quantitative non-randomised
1	Is there an adequate rationale for using a mixed methods design to address the research question?	Is the qualitative approach appropriate to answer the research question?	Is the sampling strategy relevant to address the research question?	Are the participants representative of the target population?
2	Are the different components of the study effectively integrated to answer the research question?	Are the qualitative data collection methods adequate to address the research question?	Is the sample representative of the target population?	Are measurements appropriate regarding both the outcome and intervention (or exposure)?
3	Are the outputs of the integration of qualitative and quantitative components adequately interpreted?	Are the findings adequately derived from the data?	Are the measurements appropriate?	Are there complete outcome data?
4	Are divergences and inconsistencies between quantitative and qualitative results adequately addressed?	Is the interpretation of results sufficiently substantiated by data?	Is the risk of nonresponse bias low?	Are the confounders accounted for in the design and analysis?
5	Do the different components of the study adhere to the quality criteria of each tradition of the methods involved?	Is there coherence between qualitative data sources, collection, analysis and interpretation?	Is the statistical analysis appropriate to answer the research question?	During the study period, is the intervention administered (or exposure occurred) as intended?

### Characteristics and Features of Sports Schools

Eleven studies [19, 24, 27, 28, 37, 40–45] explored sports schools' characteristics and features, and are summarised in Table 2. Sports schools are situated in upper and lower general and vocational secondary education (International Standard Classification of Education level 2–5 [19]). Data highlighted that the majority of programmes supported athletes through development (i.e., athletes narrow their focus to one or two sports) and mastery (i.e., athlete becomes an expert in their sport) phases of their athletic development [19]. Sports schools can either be an education-led or a vocation-led system (i.e., the athlete is based in an education/vocation environment that offers support for sport and performance), or a combined dual-career development environment (i.e., an organisation or institution that works in tandem with both sport and education/vocational providers to deliver an all-round support package to the individual undertaking the dual-career [19]). However, the support provision between institutions in the same country is not standardised because each is able to decide what support they provide, but they can include similar features (e.g., sports facilities, academic support, sport science provision) [19]. The thematic analysis identified four themes: academic support services, athletic support services, intense routines and training partners.

#### Academic Support Services

Academic support services within sports schools included extra tutoring to help after periods of absence [24, 42], adaptation of school and training schedules [24, 42], lighter load by one academic subject [27, 28, 42], extra tuition hours for athletes away at training camps or competitions [24, 42], an extra year of study [27, 28, 42], academic structure (e.g., timetabled lessons or study periods [27, 28, 42]), and career advice [41]. In contrast, one study showed that only 25% of non-sports school student-athletes received additional study support [41].

#### Athletic Support Services

Athletic support services within sports schools included better training facilities [41], high-quality coaches (e.g., former elite athletes head-hunted for international coaching roles [24, 42]), sports training as part of the daily school programmes [42] and additional provision and access to services (e.g., nutritionists, nurses, physiotherapists, and other support personnel to deal with issues related to their athletic career [24]). However, in one study, non-sports school athletes rated the services, the relationship with the coach, the coach's presence during competitions, and support services upon leaving school more positively than sports school student-athletes [41].

#### Intense Routines

The majority of sports school student-athletes’ routines were significantly more intense than their previous routines and schedules [37, 40, 44], with higher demands in school (e.g., 20–25 h work per week [37, 44]), more time in practice (e.g., average ten times or 20 h of intensive practice a week) and competitions [40, 44], perceived excessive training loads [43, 45], and strict training programmes of high intensity [37, 40, 43, 44] highlighted.

#### Training Partners

Findings indicated that many sports schools have quality training partners as indicated by sports school student-athletes’ “having someone to aspire to” [42], being surrounded by skilled people they can learn from and seek new knowledge [24], and having role models for the younger student-athletes [27, 42].

### Impacts

Forty-four studies (all except [19, 24]) evaluated the impacts associated with sports school programmes. The data collection methods and instruments used to evaluate impacts are presented below to gain a better understanding of the typical methods used to assess individual impacts. The data collection method/instrument and the key findings of these studies are also summarised in Table 2. Following the Holistic Athletic Career model [14], the thematic analysis included in four main themes: academic/vocational impacts, athletic/physical impacts, psychosocial impacts, and psychological impacts with sub-themes presented within each main theme.

#### Academic/Vocational

Twelve studies [22, 27, 36, 37, 40, 41, 43, 44, 46–49] explored academic and vocational impacts. The thematic analysis included four sub-themes: school experiences, school academic success, higher education success, and career success.

##### School Experiences

Seven studies explored the effect of sports schools on school experiences through interviews [36, 40], standardised questionnaires [22, 37, 48] and non-specified questionnaires [44, 49]. Missing school was a common issue experienced by numerous sports schools’ student-athletes [22, 40, 44, 49]. Finnish alpine and cross-country athletes missed on average 88 and 22 of 190 days per academic year [44], respectively. Furthermore, missing significant days of study [49], missed examinations owing to competitions [22], and missed lessons due to competitions [22] were scenarios often reported by sports school student-athletes. Although alpine student-athletes perceived that sports school helped combine sport and school [44] and football student-athletes appreciated the school routine [40], Dutch student-athletes who had attended a sports school were no more satisfied with the combination of school and sport than athletes who had attended a mainstream school [49]. Additionally, higher demands in school than before was one of the least satisfied factors by Swedish athletes [37] and most Finnish student-athletes considered school activities to be an inevitable part of youth, which consumed all their “free” time after sport [36].

##### School Academic Success

Six studies explored the effect of sports schools on school academic success through standardised questionnaires [22, 41, 44, 46, 49], interviews [41, 47], and classroom observations [40, 41, 47]. In the majority of studies, sports schools did not impact upon the attainment of diplomas [41], grade point average [46], secondary school qualifications [22], high school graduation [22], and high academic achievement [47]. However, Dutch student-athletes who attended a sports school attained lower educational levels in their secondary school education than student-athletes who attended a mainstream secondary school [49] and 73% of Finnish alpine skiers felt that sport participation negatively affected their success in school [44].

##### Future Higher Education Success

Five studies explored the impact of sports schools on future higher education success through non-specified questionnaires [22, 41, 49], interviews [27, 40, 41], field notes and observations [27]. Sports school student-athletes often developed competencies (such as commitment and time management) that could lead to a university athletic scholarship and future career [27]. The results regarding higher education continuation were mixed, with De Bosscher et al. [41] revealing no significant difference in continuation to higher education between student-athletes within and outside sports schools, yet in other studies, sports school student-athletes were less likely to start higher education [40, 49]. Swedish sports school student-athletes who did continue to higher education had lower higher education attainment [49] compared with mainstream school students.

##### Career Success

Two studies explored sports schools' effect on career success through a standardised questionnaire [22] and an interview [43]. German sports school student-athletes had greater likelihood of joining the Army or national police force than non-sports school pupils [22]. Czech Republic sports school student-athletes had only a limited experience with “ordinary” life outside competitive sport and therefore, reported only a minimal sense of agency about other possible professional careers and had difficulties in finding a new direction in life [43].

#### Athletic/Physical

Twenty-seven studies [20, 22, 23, 27, 36, 40–42, 44, 45, 48–64] explored athletic and physical impacts. The thematic analysis shaped four sub-themes: physical and physiological development, performance success, health and wellbeing, and drop-out.

##### Physical and Physiological Development

One study [64] using physical and physiological assessments/analysis explored the impact of a sports school on physical and physiological development of swimming and racket sports athletes. Over the 2-year investigation, haemoglobin and vital capacity linearly increased, reflecting a sports-specific response to training, but also the effect of testosterone production during the onset of puberty [64]. The resting heart rate (HR) remained on the same level and dynamic back strength increased over the two years until it plateaued during the last half-year in the swimming group [64].

##### Performance Success

Six studies explored the impact of sports schools on performance success through non-specified questionnaires [41, 44, 49], standardised questionnaires [22], interviews [20, 41, 42], participant observation [20, 42] and document analysis [20, 42]. The majority of sports schools have not led to marked differences or increases in the number of student-athletes performing at the world level as indicated by no significant differences in the performance levels and highest level reached between student-athletes within and outside sports schools [41, 44]. Only 40% of the alpine skiers and 62% of the cross-country skiers were satisfied with their present athletic success [44]. However, one study found that sports school student-athletes demonstrated higher top place finishes [41] and more medal success [41] compared to mainstream school student-athletes. On the other hand, another study [22] found no significant differences between medals won by sports school and non-sports school student-athletes.

##### Health and Wellbeing

Twenty-one studies explored the impact of sports schools on health and well-being, through interviews [27, 36, 40, 55, 59], clinical analysis [53], laboratory tests [61], non-specified questionnaires [23, 45, 50, 53, 58, 61–63], standardised questionnaires [26, 48, 51, 54, 56–58, 60], and male and female silhouettes [60].

Sports school student-athletes indicated a high incidence of injury [48, 58] and illness [58]. Clinical diagnosis of jumper’s knee together with structural changes and neovascularisation in the tendon were found to be more common among Swedish elite junior volleyball players who had attended a sports school compared to controls [53]. Sports school student-athletes felt physically ill-prepared for the intensity of sports school programmes [40] and pushed themselves so much that they sustained injuries [45, 59]. Further, sports school drop-outs complained significantly more often about physical symptoms than non-dropouts [50]. In terms of illness incidences, sports school student-athletes reported an average of 3.6 health problems per person during a 26-week period [58], a higher proportion of self-reported physician-diagnosed asthma than controls [52], and a high incidence of iron deficiency and iron-deficiency anaemia [61].

On the other hand, sports school student-athletes often demonstrated stable levels of general health and well-being [23, 62], lower levels of depression and anxiety, and excessive weight and shape concerns [63], spent more time in sport and less time in sedentary activities (screen-based behaviours) [23, 36] and smoked to a lesser extent than the reference group [61]. Furthermore, the results suggested that disordered eating is less of a problem among sports school student-athletes than in the community. Significantly more non-athletes reported dieting [56, 60], being underweight [60], eating behaviour disorder [60], being "at-risk" for eating disorders [55] and use of pathogenic weight-control methods [56] compared to sports school student-athletes.

##### Drop Outs

Two studies explored sports schools' effect on drop out through a standardised questionnaire [22] and demographic and sport-related data [50]. Both studies showed a high number of drop-outs from sports schools, with 629 pupils from 27 sports schools dropping out before attaining a school qualification [22], and 29.6% of sports school student-athletes terminating their sports careers prematurely but still pursuing their academic education [50].

#### Psychosocial

Nine studies [20, 27, 37, 42, 47, 59, 65–67] explored psychosocial impacts. The thematic analysis included four sub-themes: social skills, higher social status, family and friends, and life skills.

##### Social Skills

Two studies explored the effect of sports schools on social skills through a standardised questionnaire [47], interviews [42, 47], and observations [42]. Data revealed that social skills (skills used to interact and communicate with others) was one of main areas wherein sports school student-athletes showed individual development [42]. Furthermore, sports school student-athletes tended to rate themselves significantly higher in the social domain than non-athletes [47].

##### Higher Social Status

Two studies explored sports schools' effect on social status, through interviews, field notes/observations, and document data [20, 27]. In the two studies, the coaches classified the majority of sports school student-athletes as successful in sports, work ethic and discipline, and as “role models” [20, 27]. Classifying students as high achievers, elite, motivated, strong, competitive, and as “the really good people” and distributing them into sports schools was seen to perpetuate an elitist discourse that positioned elite athletes as having status, popularity, and recognition [20, 27]. However, this also caused tension amongst those within the programmes who received little recognition [27].

##### Family and Friends

Four studies explored sports schools' impacts on family and friends, through interviews [42, 65–67], and documents, letters, and observation [65]. Time away from family and friends outside of sport seems to be a typical consequence for student-athletes in sports schools. Results showed that many sports school student-athletes had less time for peers outside of sport [42, 66, 67] and continually negotiated the terms of their membership of that group, for example, attending activities less frequently [67]. However, several sports school student-athletes spent a great deal of time together in class, training, competitions, living and leisure, and pursuing a common career goal that tended to result in friendships/relationships arising along the way [42, 65]. Competitions were highlighted as important social events, where athletes from different clubs and nations met, socialised, and made many friends [42].

##### Life Skills

Three studies explored sports schools' impact on life skills through in-depth interviews [37, 42, 59] and participant observation [42]. The results suggested that sports schools encouraged student-athletes to develop qualities and skills applicable not only in sport but also in other spheres of life, such as independence. Sports school student-athletes often organized their living (e.g., to calculate their budget), took care of themselves (e.g., washing, cleaning, cooking [37]), developed skills to manage and organise time [59] and established social skills, autonomy, responsibility, and a strong work ethic, which are helpful to them in both sport and life. [42].

#### Psychological

Twenty-one studies [18, 22, 23, 28, 37, 40, 42, 43, 45, 46, 49, 59, 63, 68–75] in total explored psychological impacts. The thematic analysis shaped five sub-themes: perceived pressure and anxiety, motivation, identity and orientation, self-optimisation and burnout.

##### Perceived Pressure and Anxiety

Seven studies evaluated the impact of sports schools on perceived pressure and anxiety measured through standardised questionnaires [22, 37, 63, 68], interviews [37, 40, 43], and a mixed methods survey [28]. Findings suggested that sports school student-athletes experienced a (perceived) inability to meet the athletic and performance requirements [22, 43], pressure to “perform well” [37] and a constant pressure and expectation to achieve from others, such as parents and teachers [43]. Balancing sport and school was often seen as (organisationally) stressful by young sports school student-athletes [28, 40]. However, female sports school student-athletes showed significantly fewer panic symptoms, post-traumatic stress, and specific phobia than female non-athletes [68]. Furthermore, a higher proportion of ordinary school students than sports school students reported low self-oriented perfectionism with high perfectionistic concerns associated with higher anxiety levels [63].

##### Motivation

Four studies evaluated the impact of sports schools on motivation through non-specified questionnaires [37, 45, 49] and interviews [42]. The dual-motivated pattern (characterised by high value placed on both school and sport) was most typical among Finnish sports school student-athletes [46]. However, the percentage of student-athletes demonstrating this pattern decreased over time at the sports school, and the percentage showing a low academically motivated pattern increased [46]. Swedish student-athletes who had attended sports schools were significantly less motivated to do well at school than their counterparts at mainstream secondary schools [49] and 51% of Finnish student-athletes stated that they could not be bothered to invest the time and energy necessary to reach the elite performance level in their sport [45]. In contrast, Henriksen et al. [42]’s study highlighted a strong work ethic/motivation as one of the main areas wherein sports school student-athletes showed individual development.

##### Identity and Orientation

Two studies evaluated sports schools’ impact on identity and orientation through a standardised questionnaire [72, 73]. The most common profile (typical for 77% of student-athletes) was a dual identity, that is, student-athletes who reported strong identification with both athlete and student roles [73]. Furthermore, sports school student-athletes were more inclined towards task orientation [73].

##### Self-Optimisation

One study using a standardised questionnaire [69] evaluated sports schools' impact on self-optimisation. Sports school student-athletes compared to students of a regular school showed higher values in self-optimisation and stayed at this higher level during the three-year study [69]. A comparison concerning the living situation showed a more positive development in self-optimisation for those athletes living on campus [69].

##### Burnout

Eight studies evaluated the impact of sports schools on burnout via interviews [43, 59], non-specified questionnaires [23] and standardised questionnaires [18, 70, 71, 74, 75]. At the beginning of the upper secondary sports school, most student-athletes experienced very low levels of burnout [18, 23]. However, these student-athletes may be prone to develop more severe burnout symptoms across the later school years, indicated by an increase in sport and school burnout scores of the sports school students over time [70, 75]. Sorkkila et al. [74] found that sport and school burnout dimensions remained relatively stable during the first year of upper secondary school.

## Discussion

**Fig. 2:**
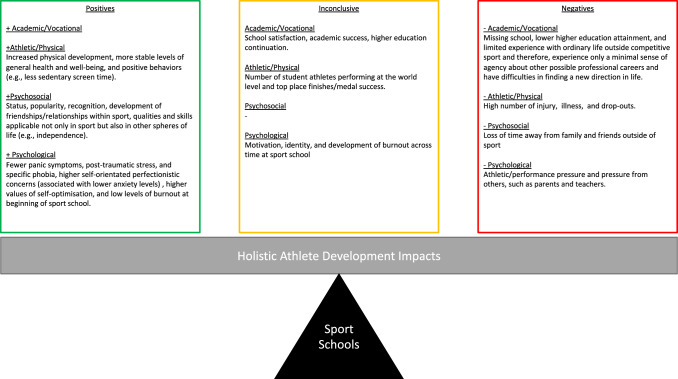
Summary of the positive and negative holistic impacts associated with sport schools

This mixed methods systematic review is the first to (1) determine the characteristics and features of sports school programmes; (2) identify the methods used to evaluate sports school holistic athlete development impacts; and (3) evaluate the impacts on holistic athlete development associated with sports school programme involvement. In total, 46 studies were identified that included 11 studies determining the characteristics and features of sports school programmes, and 44 studies that evaluated the impacts on holistic athlete development associated with sports school programme involvement. In summary, the systematic review identified the majority of research designs were quantitative non-randomised and were conducted within Northern European countries (e.g., Denmark, Norway, Sweden, Finland). Overall, the systematic review identified (1) sports school athletes receive considerably more support in academic and athletic services, more time in training and competitions, have higher-level training partners and miss more days of school than athletes outside sports school programmes; (2) a large range of data collection methods and instruments were used within the literature to evaluate a wide variety of impacts; whilst insightful from an individual study perspective, this means that impacts were often only investigated within a single or small sample of studies, thus making generalisable conclusions regarding impact difficult; (3) there are a multitude of immediate, short- and long-term positive and negative impacts (see Fig. 2) associated with being a sports school student-athlete that stakeholders (e.g., teachers, coaches, schools, parents, students) should be aware of when designing, implementing, and evaluating sports school programmes.

### Literature Methodology

This study specifically aimed to understand study methodology and data-collection methods in order to enable an evaluation of the quality of the current literature, which is pivotal in establishing the strength of the existing evidence as well as guiding future research. Overall, for all research designs the average quality assessment was above 4. This suggests that on average methodological quality was high with sufficient methodological detail provided (e.g., blinding, enrolment rates, drop-out rates, control for confounding variables) and strong philosophical or theoretical underpinnings. However, the standard deviations are quite large for such a small quality assessment range (i.e., 0–5), reflecting the variation in study quality across the studies. Addressing quality in mixed methods designs can be more difficult than in monomethod studies, due to the greater complexity of the former [76]. From the 46 studies included in the systematic review, 11 studies evaluated characteristics and features of sports schools and 44 evaluated impacts across holistic athlete development (i.e., academic/vocational, *n* = 12, athletic/physical, *n* = 27, psychosocial, *n* = 9, and psychological, *n* = 21). This demonstrates a reasonably balanced examination of holistic athlete development in the literature, although the academic/vocational and psychosocial domains have been explored less frequently compared to the athletic/physical and psychological domains. In addition, most studies were uni- or bi-dimensional. For example, only 11 studies examined two holistic impact themes, only seven studies examined three themes and no studies examined all four areas of holistic athlete development [14]. This often has to do with researchers working within specific research areas (i.e., physical vs. psychological). Therefore, future research needs a more interdisciplinary approach, which has been rare.

When analysing across the four main themes of holistic athlete development, multiple subthemes were identified, demonstrating further breadth of evaluation. Equally, when considering the methods used to assess impacts, these were highly variable with most utilising interviews (*n* = 15) and questionnaires (i.e., standardised, *n* = 19; non-specified, *n* = 12) alongside multiple other methods (e.g., observation, field notes, clinical analysis). It is a strength of this mixed methods systematic review that it is able to combine multiple methodologies in understanding the impacts of sports schools. However, as the existing research is highly variable in terms of impact area and the method used, this makes comparisons across studies to develop consensus on the impacts of sports schools difficult but does offer multiple avenues for future research. Furthermore, in the current evidence base, there is a lack of research evaluating a single enterprise or context (programme evaluation projects) of a sports school. Finally, most research-designs use self-report measures which have limitations (e.g., response bias, honesty, introspective ability, misinterpretation of questions and sampling bias). Most experts in research suggest that self-report data should not be used alone, as it tends to be biased [e.g., 77]. Research is best done when combining self-report data with other information (e.g., individual’s behaviour or physiological data). Therefore, future sports school research should adopt a “multi-method” research design to provide a more comprehensive picture of the holistic impacts of sports school programmes.

### Characteristics and Features of Sports Schools

In terms of the characteristics and features of sports schools, our findings highlighted that many sports schools offered student-athletes considerable academic and athletic support (e.g., high-quality coaches, physiotherapy, lighter load by one subject and adaptation of school and training schedules) [24, 28, 41, 42]. Whilst non-sports schools may offer time off to practice and might adapt the school day, they usually are not able to offer the same range of support services as sports schools [78] due to available resources (e.g., finances). Furthermore, sports schools may also have an advantage over club-based development as they are better able to manage the competing calls on the student-athletes’ time [24] due to resource economic efficiency (e.g., limited travel time, everything on site, extra support services, and a more flexible system). However, those outside sports schools who received extra services often rated such services better [41]. Therefore, although sports schools appear to provide considerably more support services than non-sports schools, the services available may not be as high quality, which may be an area for development within sports school programmes.

Along with additional athletic and academic support services, sports school programmes often offer high quality training partners, role models [24, 27, 42], and more intense training and competition routines [37, 40, 43]. For the welfare and well-being of student-athletes at sports schools, training and competition workload should be appropriately monitored and assessed. Without careful planning and monitoring, student-athletes are at an increased risk of excessive training loads, insufficient rest and recovery [79–83], injury [84–87], and burnout [88, 89]. While overtraining and non-functional overreaching are not exclusively a consequence of training overload, it is likely that sports schools that fail to provide sufficient recovery time for adaptation and natural growth will increase the chances of negative health impacts in youth athletes [79].

Although many characteristics and features of sports schools are highlighted above, there are common themes that have been identified in talent identification and development systems (TIDS) that have not been reviewed in the current sports school literature. We are aware that training within sports schools can be intense and competitive, but little is known about what the training at sports schools involves. For example, according to Ko et al.’s review [90], there are many trainable factors contributing to sport success (i.e., physiological variables, psychological attributes, physical performance, sport skills). Most training programmes, however, focus only on physical skills, although psychological variables have been identified as critical determinants of sporting development and for the maintenance of excellence [91]. Future research should explore in more detail the context of training within sports schools to assess whether they offer well-rounded, holistic development programmes. In addition, with the knowledge that training programmes at sports schools may be intense and competitive, little is known about the recovery strategies used within these sports school settings/contexts. Martindale and Mortimer’s [92] review suggested that effective emotional/physical recovery needs to be emphasised in youth sport programmes in order to prevent injury and avoid other negative psychological consequences (e.g., stress and burnout). Various strategies with regard to training load, nutrition, cooling down, stretching, social support, and lifestyle education (e.g., time management and planning) can be included in a multidimensional training programme in order for the athletes to achieve the balance between life and training [92, 93]. Therefore, as injury has been highlighted as a common issue within sports schools, future research could evaluate the training and recovery strategies/methods of sports school programmes to maximise holistic athlete development.

### Impacts

Given the increased popularity of sports schools, the fact that only a few athletes ever obtain a professional status, and the multiple positive and negative impacts associated with intensified youth sport programmes, understanding the impact of sports schools on holistic athlete development is important to inform their design, implementation, monitoring and evaluation. The combination of sport and academics is a key aspect of sports schools. However, current findings suggest contradictory impacts across sports school programmes across different countries and sports. Missing school was a common impact experienced by sports schools’ student-athletes [22, 40, 44, 48]. However, although student-athletes missed schoolwork, four (out of six) studies [22, 41, 46, 47] showed that attending a sports school did not impact upon school academic success. These findings are congruent with past research, which has indicated that elite athletes achieve in both sport and school [94–96]. Such findings within sports schools may reflect the importance of additional support offered by sports schools (e.g., extra tutoring, adaptation of school and training schedules, lighter load by one subject, extra year of study) in protecting academic success. Therefore, whilst sports school athletes may miss periods of school work, strategies are in place to overcome such negative impacts.

Whilst the evidence supports the fact that sports school athletes were not negatively impacted in the short term, there may be more longer-term negative implications. The findings on higher education continuation were mixed [40, 41, 49], with some sports school student-athletes achieving lower higher education attainment [49] compared to mainstream school student-athletes. Therefore, although student-athletes tended to display a dual identity while at sports schools [72], the fact that more athletes outside sports schools attained higher education grades may indicate that sports school student-athletes choose to prioritise their sport over their studies to a greater extent once they leave the sports school and their sporting careers progresses. An overemphasis on sport may pose issues in terms of career opportunities for elite athletes once their athletic careers end. Therefore, although evidence suggests that sports schools provide adequate support for student-athletes to pursue both education and sport [72], little is known about the consequences of combining elite sport and subsequent career success. Some studies have shown that top athletes obtain higher ranking jobs than non-athletes [97, 98]; however, further research is needed to establish the specific impact of attending a sports school on student-athletes’ future development outside their athletic careers [98].

Alongside academic development, the aim of a sports school is to develop athletic and sporting performance. Whilst one study demonstrated physiological and physical development across sports school training programmes [64], research suggested sports schools have not resulted in an increased number of student-athletes performing at the world level [41, 49]. Together with findings on top place finishes and medal success results being mixed [22, 41, 49] this raises concerns about the effectiveness and importance of specialist support and training within sports schools. In this respect, the fact sports schools have not led to marked differences in the number of student-athletes performing at the world level should not automatically lead to the conclusion that sports schools are not suitable for facilitating the combination of elite sport and education. There are multiple confounding factors influencing sporting success, such as genetic qualities of young talents and their close environment at the micro-level (e.g., parents and friends [37, 99, 100]), organisational and policy factors at the meso-level (e.g., sport clubs, international competitions and scientific research and innovation [100–102]), and factors at the macro-level (e.g., media, sponsorship, politics, school system, geographic factors and performance culture [37, 99, 100]). Ultimately, sports schools can only ever be somewhat successful due to limited spaces at the top of the pyramid. However, if the majority of other impacts are positive and athletes (at the same rate as in other TIDS) make it to the top, they are probably successful. As such we may also need some comparative numbers (through future research) from non-sports school contexts to appreciate what ‘normal’ performance success would be. It is also important to consider the bigger picture (holistic advantages) of sports schools (i.e., not just focusing on sport performance). As a whole, attending a sports school might be the only possibility for young student-athletes to combine school and elite sports. Therefore, even though sports schools may not guarantee better sporting performances and academic success, without sports schools, student-athletes may not be able to pursue their sporting ambitions at all, or they might have been even less successful in school/sport or might have become school/sport drop-outs. Overall, due to the sports school set up, there is hope for added value (i.e., instead of allowing youngsters to pursue elite sports away from school and not make it, and sacrifice their schooling, here they may at least safeguard their schooling and have a more pleasurable/balanced experience along the way).

It is impossible to eradicate all injuries from youth sport programmes; however, injury prevention schemes that develop appropriate training can significantly reduce the frequency and severity of injuries [103]. Consequently, since many student-athletes were at a high risk of becoming injured after enrolment into sports schools, appropriate recovery and prevention strategies should be incorporated as part of sports school programmes. These could include strength and conditioning (S&C) programmes focused upon strength, endurance, and proprioception/balance [104–106], collaboration and communication with stakeholders on managing youth athlete training schedules [107], monitoring of individual workload [107], modifying external training variables to achieve a desired internal response [107], athlete education [107], extrinsic factors via the use of protective equipment (e.g., ankle bracing and taping, helmets, and mouth guards) [103, 108] and implementation of rules and regulations [85, 109].

Sports schools attempt to help athletes achieve athletic success at an early stage. As a result, training is intensified. An unintended consequence is an increased risk of early performance stagnation and thus higher number of drop-outs from sport and sports schools [22, 50]. Consistent with earlier studies [110–112], Baron and Alferman [50] demonstrated that physical complaints (e.g., injury, fatigue, illness), motivation, and volitional skills are important predictors of sports school dropout. Furthermore, the development of psycho-behavioural skills (e.g., time management skills, effective communication, social awareness, and maturity) has been shown to support transition to new environments after deselection [113]. This study supports the notion that sports schools contributed to the development of student-athletes’ social skills [42, 47] and life skills (e.g., independence and time management [37, 42, 59]), while being more inclined towards task orientation [73], which is associated with positive self-image, satisfaction and high performance in sports [114, 115]. As such, it may be suggested that the best foundation for noticeable, permanent development within, through and after sports schools would be a focus on developing and supporting personal motivation, task-orientation, and volitional and psycho-behavioural skills, while reducing physical complaints for every individual at any stage of participation [113].

Being identified as talented can change the nature of peer relationships [3]. Although many sports schools positioned student-athletes as having positive status, popularity and recognition [20, 27], this also caused tension amongst some student-athletes within the programmes [27]. Furthermore, although sports schools may have provided many opportunities to retain and develop friendships within sport, time away from family and friends outside of sport was a typical consequence for student-athletes [42, 45, 66]. Researchers have highlighted the risk of social isolation and feelings of alienation that result from spending substantial amounts of time away from family and inevitably having fewer opportunities to make and retain friendships outside sport (e.g., [116, 117]). The reduced ability to form non-sport friends could result in a lack of social support when athletes terminate their athletic career and are potentially isolated from their friends within sport [118]. Furthermore, previous studies have demonstrated that a strong athletic identity was negatively associated with the quality of athletes’ career transitions [119, 120]. Therefore, given that all athletes eventually have to transition out of sport, it would be worth exploring if these negative impacts apply to sports school athletes that showed both a strong student and athletic identity [72].

Combining an athletic career with education is demanding for student-athletes [121], and junior athletes are susceptible to stress and burnout (e.g., [122, 123]). When starting at a sports school, most student-athletes experienced very low levels of burnout [18, 23]. However, the results on the development of burnout across time at sports schools were mixed. These findings suggest that among student‐athletes at sports schools, there are different subpopulations with different developmental trajectories, such that in some student‐athletes symptoms of burnout may increase, whereas in others, the symptoms remain relatively stable. This highlights the importance of continuous screening (e.g., profile of mood test) and early detection of burnout in student-athletes at sports schools [18, 70, 75]. Furthermore, this also suggests the need for careful management of the performance environment as concerns have been raised that youth athletes are increasingly being exposed to inappropriate and unrealistic demands and expectations, resulting in psychological overload [80]. Indeed, some sports school athletes experienced athletic/performance pressures [22, 31, 37, 67] as well as pressure from others, such as parents and teachers [43]. Adopting a dominant performance focus can lead to high levels of perceived pressure, feelings of low self-esteem and confidence [124] in addition to a fear of failure associated with the risk of being evaluated negatively and letting down significant others [125]. TIDS research has highlighted that parents, coaches and peers have the potential to promote a “winning at all cost” mentality, or adopt particular behaviours in response to failure and in search of better results, such as pressing athletes to ‘push harder’ [126, 127]. Such pressure can contribute to an unhealthy training environment [126, 127] and will likely require careful management in sports schools.

Although being a student-athlete at a sports school may be associated with more pressure, training demands and expectations, current findings highlighted the important influence sport within sports schools may have upon student-athletes' mental health, health behaviours and their willingness to shift their time away from unhealthy behaviours related to general health and wellbeing. Sports school student-athletes spend a considerable amount of time engaging in sport/physical activity. Consequently, it is unsurprising that numerous sports school student-athletes demonstrated more favourable levels of general health and well-being [23, 62], fewer mental health related symptoms [62] and more protection against unhealthy and risky behaviours [23, 47, 61]. This is in line with previous research that has shown positive health benefits [128–130] and lower rates of unhealthy and risky behaviours (e.g., less screen time, smoking, drug use) with increased physical activity and participation in sports [131, 132].

Overall, there are many different characteristics and features of sports school programmes, which can be implemented in a variety of ways. As a result, impacts are likely to vary across every sports school context. The success of sports schools often depends on many situational factors, such as financing, goodwill of the person in a key position of an organisation, quality of coaching and teaching staff involved, and culture [37] affecting whether sports schools provide benefits and positively contribute to school-age athletes’ holistic development [37]. It is not possible for this systematic review to establish a rigorous causal relationship between the characteristics and features of sports schools and the associated impacts. This means that more studies exploring the characteristics and features of such sports schools and how these relate to impacts are needed to account for the socio-cultural context and local conditions of programmes [37]. Equally, little is known about the motives and reasons why sports school student-athletes attend sports schools, warranting future research. Overall, it was evident that involvement with sports school programmes is associated with a range of potential positive and negative impacts (summarised in Fig. 2). Strategies should be put in place that emphasise the holistic development of youth athletes and that try to mitigate the negative and promote the positive impacts associated with sports schools to ensure system "worth" [8]. It has been suggested in TIDS research that positive impacts emerge from higher quality TIDS [8]. The same concept could be related to sports schools, where the issue does not lie with the overall concept of sports schools, but instead, their impact reflects how well they are designed, implemented and managed [8].

### Limitations of Existing Research

Whilst the current systematic review highlighted the breadth of impacts associated with sports school programmes, a number of limitations exist within the current evidence base. Firstly, as stated above (Sect. 4.1), most studies included in this review are uni- or bi-dimensional. Whilst studies examine individual components of holistic athletic development, no sports school research evaluates all four areas (i.e., educational/vocational, physical/athletic, psychosocial and psychological). Therefore, more multi-dimensional studies assessing the holistic development of student-athletes at sports schools are warranted. However, although the Holistic Athletic Career Model has been used extensively in previous research studies within sport to guide data collection about the athlete as a whole person (e.g., [37]) within the current study it may be more useful as a guiding conceptual framework due to the lack of empirical examination and testing within sports school contexts.

Interestingly, the majority of studies (72%) have been conducted in northern European countries (i.e., Denmark, Norway, Sweden, Finland), especially those countries that have state sponsorship and specific policy approaches toward the dual-careers of student-athletes [133]. It is important to recognise cultural, social and policy factors differing across countries and sport settings, which challenges the generalisability of the findings in this study. It is difficult to apply current research on sports schools' effectiveness to other countries as each country may have its own systems and approaches [26]. Sports schools may vary in their resources, organisational structure and aims/objectives, which are likely to affect whether sports schools provide benefits or contribute to school-age athletes' holistic development [37, 100]. This means that exploring the impact of sports schools across different countries is warranted to account for the socio-cultural context and local conditions of dual-career programmes. In addition, the data in this study cannot be generalised across sports, but rather require a sport-by-sport analysis. It is expected that sports schools require an individual approach, tailor-made for each athlete and each sport. Therefore, future research needs to take the specificity of athlete characteristics/variables (e.g., sex, type of sport, age, development stage within the school, training cycle) into account.

Finally, it is not possible to establish a rigorous causal relationship between attending a sports school and the impacts established in this study. The study designs within this systematic review are unable to evaluate whether impacts are a direct result of the sports school or any other confounding factors (e.g., genetic qualities, being an athlete, parents, friends, sports clubs, international competitions, media, sponsorship, politics, geographic factors, and performance culture, etc. [100]). Furthermore, there is a lack of studies utilising a control or comparative group design within the current sports school literature. It is important for us to explore in future research if sports schools through combining schoolwork with an intensified and competitive sport regime offer a return on investment that goes beyond what is to be expected at non-sports schools. Only three studies [22, 41, 49] compared student-athletes within a sports school directly with student-athletes outside a sports school. These three studies [22, 41, 49] all used a retrospective approach and design. This approach could lead to incomplete or inaccurate information due to selective memory loss (recall bias) or to participants' social desirability to describe the dual-career development in a more positive light. The studies also all used one single measurement, namely an online survey or interview. This opposes the nature of “transition” as a process, which calls for using a longitudinal approach to investigate student-athletes' development or changes over time. Therefore, more studies comparing student-athletes within a sports school directly with student-athletes outside a sports school, as well as longitudinal studies that multidimensionally examine the impacts of sports school involvement in real-time and as they occur across the athletes’ development, are warranted.

## Conclusion

This systematic review prompts a debate and critical reflection about (1) the characteristics and features of sports school programmes; (2) the methods used to evaluate sports school impacts; and (3) the positive and negative impacts on holistic development associated with sports school programme involvement. A range of characteristics and features of sports schools (e.g., athletic and academic support services) were identified; however, further research is needed to gain a more in-depth understanding of how these characteristics and features are operationalised across different contexts as well as how they relate to impacts. A large range of data collection methods and instruments were used within the literature to evaluate a wide variety of impacts; whilst insightful at study level, as a result, specific impacts are often only studied in a single or small number of studies and few studies are truly holistic or multidimensional in nature. This makes comparison across studies and developing consensus on the impacts of sports schools difficult. Therefore, more multi-dimensional and longitudinal studies assessing the holistic development of student-athletes at sports schools in “real time” are required. Furthermore, more information on the motives and reasons why sports school student-athletes attend sports schools is needed.

Nevertheless, from the current literature there are a multitude of immediate, short- and long-term positive and negative impacts associated with being a sports school student-athlete that stakeholders (e.g., teachers, coaches, schools, parents, students) should be aware of. The positive impacts included increased physical development, more stable levels of general health and well-being, positive behaviours, status/popularity, development of friendships within sport, life skills, higher values of self-optimisation and low levels of burnout in the initial phases of joining a sports school. The negative impacts included missing school, lower higher education attainment, limited experience with ordinary life outside of competitive sport, high number of injuries, illness and dropouts, loss of time away from family and friends outside of sport, performance pressure and pressure from others (e.g., parents and teachers). Practitioners should be aware that they can promote (positive) and negate (negative) health impacts through the design of an appropriate learning environment that simultaneously balances multiple training (e.g., load), academic (e.g., exams), psychosocial (e.g., sense of community), and psychological (e.g., identity) factors that can be challenging for youth athletes [1, 2]. To aid careful management, practitioners should aim to design and implement monitoring and evaluation tools that assess the holistic development of student-athletes within their sports schools. Such monitoring tools could assess a range of factors including athlete wellbeing [83, 134], training load [135], physical development [136] and injury prevalence [137], alongside psychosocial factors (e.g., athletic identity [138]), education [2] and long-term health and performance development [8]. In summary, sports schools seem to be a potentially beneficial strategy for athletes to combine their pursuit of a sports career with education and other domains of life (e.g., social life). However, it is important to understand and mitigate against the negative impacts observed in such programmes to ensure healthy and holistic athlete development.

## Declarations

### Availability of data and materials

The datasets generated during and/or analysed during the current study are available from the corresponding author on reasonable request.
